# Far-Red Fluorescent Proteins: Tools for Advancing In Vivo Imaging

**DOI:** 10.3390/bios14080359

**Published:** 2024-07-24

**Authors:** Angyang Shang, Shuai Shao, Luming Zhao, Bo Liu

**Affiliations:** 1 Cancer Hospital of Dalian University of Technology, Shenyang 110042, China; anyashang@mail.dlut.edu.cn (A.S.); shaos@dlut.edu.cn (S.S.); zhaoluming@dlut.edu.cn (L.Z.); 2 Liaoning Key Lab of Integrated Circuit and Biomedical Electronic System, Faculty of Medicine, Dalian University of Technology, Dalian 116024, China

**Keywords:** far-red fluorescent proteins, fluorescent biosensors, fluorescence imaging

## Abstract

Far-red fluorescent proteins (FPs) have emerged as indispensable tools in in vivo imaging, playing a pivotal role in elucidating fundamental mechanisms and addressing application issues in biotechnology and biomedical fields. Their ability for deep penetration, coupled with reduced light scattering and absorption, robust resistance to autofluorescence, and diminished phototoxicity, has positioned far-red biosensors at the forefront of non-invasive visualization techniques for observing intracellular activities and intercellular behaviors. In this review, far-red FPs and their applications in living systems are mainly discussed. Firstly, various far-red FPs, characterized by emission peaks spanning from 600 nm to 650 nm, are introduced. This is followed by a detailed presentation of the fundamental principles enabling far-red biosensors to detect biomolecules and environmental changes. Furthermore, the review accentuates the superiority of far-red FPs in multi-color imaging. In addition, significant emphasis is placed on the value of far-red FPs in improving imaging resolution, highlighting their great contribution to the advancement of in vivo imaging.

## 1. Introduction

Fluorescent proteins (FPs), originally derived from marine organisms such as jellyfish, coral, and anemone, have significantly enhanced the capabilities of life science research. They have introduced an array of innovative fluorescence-based approaches for elucidating fundamental mechanisms and addressing application challenges in biotechnology and biomedical fields [1,2,3]. The exploration of diverse FPs has persisted for over six decades since Osamu Shimomura isolated green fluorescent protein (GFP) from the jellyfish *Aequorea victoria* in 1962 [4]. The discovery of GFP color variants and homologs across various species has broadened the fluorescence palette of available FPs, spanning a wide spectrum from blue to red, thereby offering substantial potential for multi-color imaging [5,6,7]. FPs have become indispensable tools in reporting gene expression, monitoring protein dynamics, and observing cell behavior within complex tissues and organisms [8,9,10]. Moreover, FPs have served as fundamental templates for the development of genetically encoded biosensors. These biosensors are designed for detecting small molecules, biochemical compounds, and environmental changes under complicated biological conditions [11,12,13]. Nonetheless, traditional FPs, especially those with shorter emission wavelengths, encounter notable limitations, including interference from autofluorescence, light scattering and absorption by hemoglobin, and inherently limited tissue penetration. These drawbacks have constrained the reliability of FPs as tools in biological applications. Therefore, FPs with further red-shifted emission spectra are highly desirable as they can circumvent these challenges and provide enhanced imaging quality in vivo. Indeed, far-red FPs are particularly promising, offering enhanced resolution and deeper tissue penetration. These attributes uniquely position far-red FPs as unparalleled tools in biomedical and biotechnological applications.

Far-red FPs are characterized by red-shifted emission peaks ranging from 600 to 650 nm [14]. These proteins exhibit significant advantages for non-invasive in vivo imaging applications, particularly in deep-tissue and whole-body imaging, due to their deep tissue penetration capabilities, reduced light scattering and absorption, enhanced autofluorescence resistance, and diminished phototoxicity effects [15,16,17]. Notably, the emission spectrum of far-red FPs lies above the absorbance profile of hemoglobin (600 nm) and below that of water (1300 nm), resulting in decreased light absorption and reduced phototoxic stress to cells [18,19,20]. Moreover, as the light-scattering intensity drops off with the wavelength increases, far-red FPs with redder emission spectra exhibit lower light-scattering cross-sections. Additionally, the minimal photon scattering and low tissue absorption of far-red light enable the deeper penetration depth of far-red FPs compared to visible light, facilitating the imaging of targets within deeper tissue [21,22]. Furthermore, autofluorescence, triggered by the excitation of endogenous molecules such as nicotinamide adenine dinucleotide (NADH) or Flavin, is significantly reduced with red-shifted excitations due to the complete separation of far-red emission wavelengths from the typical autofluorescence range of cellular components [23,24]. Unlike near-infrared FPs (650–900 nm) that often necessitate biliverdin (BV) as a natural prosthetic group, far-red FPs possess an intrinsic chromophore capable of self-catalyzation, with oxygen being the sole requisite for chromophore formation. While biliverdin IXα serves as a ubiquitous metabolic product within living organisms, evidence suggests that the extra addition of exogenous BV cofactor is necessary to achieve the optimal efficiency of near-infrared FPs [25,26,27]. Consequently, far-red FPs become a valuable tool in the visualization of biological processes within living organisms, offering substantial benefits, including lower autofluorescence, enhanced penetration depth, and reduced phototoxicity.

Various far-red fluorescent proteins have significantly advanced in vivo imaging. The evolution of these far-red FPs has consistently aimed to achieve several key improvements: further red-shifted excitation and emission peaks, enhanced brightness, accelerated maturation speed, improved photostability, increased pH stability, and maintenance of a stable monomeric structure. These properties are crucial as they determine the applications of these proteins in biological research. A higher extinction coefficient (EC) helps FPs to absorb light more effectively at a specific wavelength, thus enhancing the brightness of the emitted fluorescence signal. Proteins with higher quantum yield (QY), which quantifies the fraction of absorbed photons that result in fluorescence, emit light more efficiently. The brightness of a fluorescent protein combines its EC and QY, indicating overall efficiency in producing fluorescence signals. Higher brightness values make FPs more sensitive and easier to detect in biological research [28]. The half-life (t_0.5_) refers to the time required for a process, such as maturation or bleaching of FPs, to reach half of its maximum or initial value. A shorter t_0.5_ for maturation indicates rapid maturation post-synthesis, which is beneficial for applications requiring quick protein expression and imaging [29]. Conversely, a longer t_0.5_ for bleaching signifies greater photostability, essential for prolonged imaging sessions and reduced phototoxicity in live-cell imaging [30]. The pKa value influences the fluorescence intensity of proteins in response to pH changes. A lower pKa makes proteins suitable for pH-sensitive imaging applications [31]. The quaternary structure describes how individual protein subunits assemble to form the functional unit of the fluorescent protein complex. The monomeric structure of fluorescent proteins makes them more attractive as genetically encoded fusion tags [31]. The evolution of various far-red FPs has significantly advanced in vivo imaging, making them crucial for diverse biological research applications.

Fluorescent proteins provide precious opportunities for the non-invasive imaging of cellular processes in vivo. Although numerous reviews focusing on fluorescent proteins and fluorescent sensors are available, there is a scarcity of summarized documentation regarding far-red FPs despite their extensive utilization [32,33,34]. Far-red FPs offer superior biocompatibility compared to fluorescent dyes, ensuring enhanced stability and persistence during prolonged imaging sessions. Additionally, far-red FPs are more convenient for biological research applications compared to near-infrared FPs, which require biliverdin as a cofactor for light emission. Overall, far-red FPs possess unique advantages in biological applications. This review delves into the characteristics, applications, and biological significance of far-red FPs within living systems, providing an exhaustive exploration of their functionality and the convenience of their utilization. Initially, far-red FPs, with emission peaks ranging between 600 nm and 650 nm, are summarized according to their species of origin, including *Heteractis crispa*, *Discosoma* sp., *Entacmaea quadricolor*, *Montipora* sp., and *Porites lobata*. The evolutionary relationship, photophysical and biochemical properties, and their performance in vivo are meticulously analyzed. Subsequently, the principles of far-red biosensors and reporters engineered for detecting crucial small molecules, protein–protein interactions (PPIs), and environmental changes are explicated. Furthermore, the significant benefits of far-red FPs in multi-color imaging, in conjunction with spectrally distinct FPs in the visible and near-infrared regions, are highly depicted. Special emphasis is also placed on the pivotal role of far-red FPs in enhancing resolution within imaging modalities, including photoacoustic imaging and nanoscale imaging, underscoring their significant utility in improving visualization techniques.

## 2. Far-Red Fluorescent Proteins

### 2.1. Far-Red FPs from Reef Coral Discosoma sp.

DsRed, the first natural orange–red fluorescent protein, holds significant importance as it breaks up the traditional concept that GFP-like proteins are necessarily linked to bioluminescence [35]. Owing to its exceptional brightness and orange emission spectrum, DsRed has been recognized as an ideal template for developing various far-red FPs (Table 1).

**Table 1:** Far-red fluorescent proteins.

	Ex Peak ^a^	Em Peak ^a^	EC ^b^	QY ^c^	Brightness ^d^	t_0.5_ for Maturation at 37 °C	t_0.5_ for Bleach ^e^	pKa	Quarternary Structure	References
*Discosoma* sp.										
DsRed	558	583	75	0.79	59.25	~10 h	ND	4.7	tetramer	[29]
mRFP1	584	607	50	0.25	12.5	<1 h	6.2 s	4.5	monomer	[29]
mCherry	587	610	72	0.22	15.84	15 min	68 s	<4.5	monomer	[29]
mPlum	590	649	41	0.1	4.1	1.6 h	53 s	<4.5	monomer	[30,36]
mRasberry	598	625	86	0.15	12.9	2.1 h	15 s	<4.5	monomer	[30,36]
mGrape3	608	646	40	0.03	1.2	ND	5 s	7.0	monomer	[30]
E2-Crimson	611	646	126	0.23	28.98	26 min	ND	4.5	tetramer	[36]
*Entacmaea quadricolor*										
eqFP611	559	611	116	0.45	52.2	ND	ND	ND	tetramer	[37]
mRuby	558	605	ND	0.35	ND	2.8 h	ND	4.4	monomer	[38]
eqFP578	552	578	102	0.54	55.08	ND	ND	ND	dimer	[39]
Katushka2S	588	633	67	0.44	29.48	14 min	ND	5.4	dimer	[40]
Katushka	588	635	65	0.34	22.1	20 min	ND	5.5	dimer	[41]
tdKatushka2	588	633	66.25 ∗ 2	0.37	49.2	ND	ND	5.4	monomer	[42]
mKate	588	635	45	0.33	14.85	75 min	82 s	6.0	monomer	[30,41]
mLumin	585	630	75	0.3	22.5	76 min	327 s	5.4	monomer	[30,43]
mKate S158C	586	630	63	0.33	20.79	76 min	220 s	4.2	monomer	[30,43]
mKate2	588	630	50	0.4	20	38 min	81 s	6.5	monomer	[44]
FusionRed	580	608	95	0.19	18.05	130 min	131 s	4.6	monomer	[45,46]
Neptune	600	650	72	0.18	12.96	35 min	158 s	5.8	dimer	[30]
mNeptune	600	650	67	0.2	13.4	28 min	160 s	5.4	monomer	[30,44]
mNeptune2	599	650	89	0.24	21.36	27 min	373 s	6.3	monomer	[44]
mNeptune2.5	599	643	95	0.28	26.6	26 min	506 s	5.8	monomer	[44]
Crimson	588	617	77	0.42	32.34	14 min	49 s	4.2	dimer	[46]
Other species										
HcRed	592	645	70	0.05	3.5	59 min	ND	4.0	dimer	[47,48]
tKeima	440	616	14.5	0.22	3.19	ND	ND	6.5	tetramer	[49]
mKeima	440	620	14.4	0.24	3.456	ND	ND	6.5	monomer	[49]
plobRFP	578	614	84	0.74	62.16	ND	80 s	ND	tetramer	[28]

DsRed and eqFP578 are not far-red FPs; they are just as listed as references. ^a^ Excitation and emission maxima in nm. ^b^ Extinction coefficient in mM^−1^cm^−1^. ^c^ Quantum yield of fluorescence. ^d^ Brightness is determined as a product of the quantum yield and molar extinction coefficient. ^e^ Time (s) to bleach to 50% emission intensity, at an illumination level that causes each molecule to emit 1000 photons/s initially. ND, not determined.

The progressive modulation of DsRed toward dimer, tandem dimer, and monomer variants has been accomplished by systematically disrupting AB and AC interfaces. Among these variants, the monomer variant known as mRFP1 is particularly notable for exhibiting the most red-shifted spectra compared to other DsRed variants, with excitation and emission maxima at 584 and 607 nm, respectively (Table 1). mRFP1 is also recognized as the first true monomer fluorescent protein with far-red spectra characteristics [31]. Although mRFP1 has a lower extinction coefficient (EC), quantum yield (QY), and photostability than DsRed, its rapid maturation rate—approximately 10-fold faster—ensures its comparable brightness when expressed in living cells. Notably, mRFP1 demonstrates its enhanced stability over GFP within acidic organelles, such as endosomes, lysosomes, and autophagosomes, thus gaining prominence as a pH indicator [50,51]. Due to its monomer nature, mRFP1 shows great adaptability as a fusion tag. mrfp1-labeled cell lines can be easily distinguished from negative controls during fluorescence-activated cell sorter (FACS) analysis, supporting its utility and versatility in various biological and imaging applications [52].

**Figure 1:**
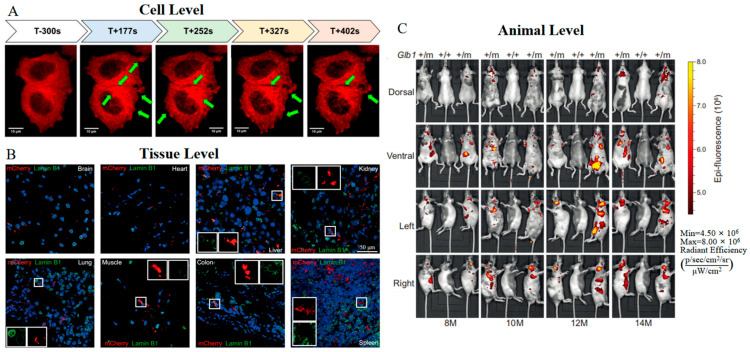
(**A**) Time-lapse images of mCherry-tubulin in MCF-7 breast cancer cells 5 min before the launch (T-300 s) of the rocket and during the real microgravity (r-µg) phase (T + 177 s−T + 402 s). The green arrows indicate changes in α-tubulin [54] (reproduced with permission from Copyright 2019, Multidisciplinary Digital Publishing Institute). (**B**) Images of Glb1-2A-mCherry in indicated tissue sections from Glb1^+/m^ mice. The white boxes show cells with mutually exclusive signals for mCherry and Lamin B1 [55]. (**C**) Images of Glb1-2A-mCherry in a cohort of Glb1^+/m^ mice at indicated ages [55]. The images in (**B**,**C**) were reproduced with permission from Copyright 2022, Springer Nature.

Based on mRFP1, mCherry was engineered with slightly red-shifted excitation and emission peaks (Table 1) [29]. Through a series of directed screenings focusing on the residues near the chromophore, the enhancements in both the maturation rate and photostability of mCherry are significant—showing improvements of 75% and 91%, respectively, when compared to mRFP1. Importantly, mCherry exhibits excellent pH stability in acidic organelles, positioning it as a viable pH indicator [53]. An additional advantage of mCherry lies in its favorable tolerance to fusions at both the N- and C-termini. This feature facilitates the generation of novel constructs, such as mCherry-tubulin and Glb1-2A-mCherry. mCherry-tubulin fusion proteins have been successfully incorporated into microtubules, thereby delineating the dynamic changes of α-tubulin in human breast cancer cells under real microgravity (r-µg) conditions (Figure 1A) [54]. Furthermore, transgenic mice expressing Glb1-2A-mCherry (GAC) remain healthy and enable the visualization of *Glb1* expression at both tissue and animal levels (Figure 1B,C) [55]. These improved properties demonstrate mCherry’s robustness and adaptability as a fluorescent marker for in vivo imaging.

**Figure 2:**
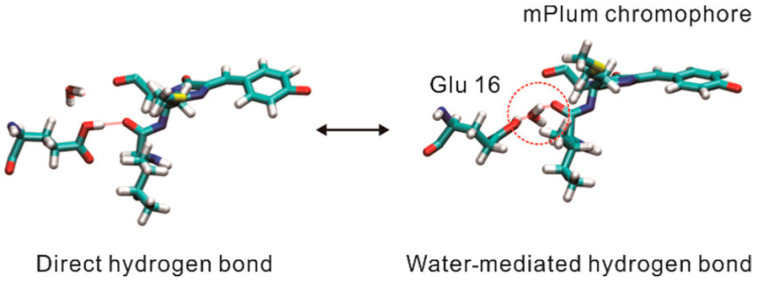
Structure diagrams of two conformations of mPlum as indicated. The water molecule inside the red circle mediates the hydrogen bonding [57] (reproduced with permission from Copyright 2022, American Chemical Society).

Building upon mRFP1, two novel variants named mPlum and mRasberry have been devised to achieve superior photostability and further red-shifted emission spectra (Table 1) [56]. With an emission peak at 649 nm, mPlum is characterized as the reddest protein among all DsRed-derived far-red fluorescent proteins. However, mPlum suffers from decreased brightness, retaining merely 7% of the fluorescence intensity of DsRed. Furthermore, the emission spectra of mPlum are temperature dependent [57,58,59]. A decline in temperature results in a spectral blue shift owing to the suppression of the excited-state interconversion between direct hydrogen bonding and water-mediated hydrogen bonding, which characterizes its emission spectrum (Figure 2). In contrast, mRasberry ensures a comparative level of fluorescence to its precursor, with a slightly red-shifted emission spectrum. However, the fluorescence of mRasberry drops off quickly as the excitation wavelength increases, falling to only 10% of peak intensity when excited at 630 nm. The intricate photophysical properties and dim fluorescence of both mPlum and mRasberry have resulted in their limited applicability for in vivo imaging.

The development of mGrape3 from mRFP1 represented an innovative approach by integrating a π-π stacking interaction near the chromophore, aimed at extending the red-shift of the excitation spectrum (Table 1) [30]. This modification makes mGrape3 characterized by dual absorbance peaks at 470 nm and 608 nm. The transition from 470 nm to 608 nm can be realized by using light within the 465–495 nm range. However, the reverse process happens slowly and generally requires the absence of light to proceed. Despite the unique advantage of being excited at wavelengths above 600 nm, mGrape3 is rarely utilized due to its complex photophysical properties, dramatically low fluorescence QY, rapid photobleaching rate, and high pKa value.

Besides mGrape3, the same π-π stacking interaction has been introduced into E2-Crimson, resulting in the excitation and emission spectra peaking at 611 and 646 nm, respectively (Table 1) [36]. The excitation peak above 600 nm qualifies E2-Crimson as a valuable fluorescent protein for super-resolution fluorescence microscopy techniques, such as stimulated emission depletion (STED) microscopy. E2-Crimson is characterized by a high EC and a moderate QY, which together contribute to its notably bright fluorescence, maintaining nearly 50% of the brightness of DsRed. The rapid maturation rate and low pKa of E2-Crimson make it attractive for the in vivo imaging of gene activities. For instance, transgenic mice expressing GFAP-E2-crimson have been employed to validate the expression pattern of GFAP through the scalps and skulls of live animals [21]. These beneficial characteristics attribute E2-Crimson as a practical tool for fluorescence imaging applications.

In brief, DsRed and its variants have significantly advanced the development of FPs by extending the spectrum of FPs into the red domain, thereby broadening the color palette available for FPs and facilitating opportunities for multi-color imaging applications. mRFP1, a monomer variant with the most red-shifted spectra, matures faster than DsRed and is particularly stable in acidic organelles, enhancing its utility in cellular imaging and as a pH indicator. Derived from mRFP1, mCherry offers a superior maturation rate, photostability, and performs well in pH stability, becoming a reliable fusion tag and pH indicator for in vivo imaging. Other variants like mPlum and mRasberry have redder emissions and enhanced photostability but face limitations in brightness and practical imaging use. mGrape3 and E2-Crimson, with excitation peaks above 600 nm, offer unique benefits for advanced imaging techniques.

### 2.2. Far-Red FPs from Sea Anemone Entacmaea Quadricolor

**Figure 3:**
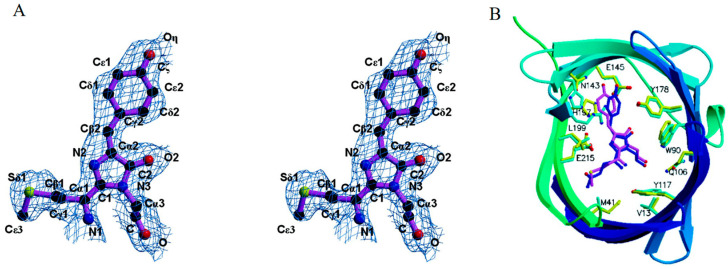
(**A**) Structure diagrams of the eqFP611 chromophore [61]. (**B**) Structure diagrams of the chromophore within the β-can fold of eqFP611. The β-can fold starts from blue, increasing to green with increasing residue number. The eqFP611 side chains are in yellow and the chromophore is in magenta [61]. The images in (**A**,**B**) were reproduced with permission from Copyright 2003, Elsevier.

With excitation/emission peaks at 559/611 nm, eqFP611 is recognized as a novel red fluorescent protein (RFP) with the most red-shifted emission spectra and the largest Stokes shift among all unmodified recombinant fluorescent proteins of the GFP family [60]. The remarkable characteristics of eqFP611, including a high EC of 116 M^−1^·cm^−1^ and a QY of 0.45, render it comparably bright to DsRed (Table 1). The crystal structure of eqFP611 has a unique chromophore sequence (Met63-Tyr64-Gly65), which is totally different from that of GFP and DsRed (Ser-Tyr-Gly in GFP; Gln-Tyr-Gly in DsRed) (Figure 3A). This unique chromophore adopts a coplanar and trans conformation within the interior of the β-can fold, explaining the far-red emission and high fluorescence nature of eqFP611 (Figure 3B) [61]. While the red fluorophore of eqFP611 exhibits much quicker maturation, it only matures at low temperatures. Moreover, the tetrameric form of eqFP611 could potentially disrupt the natural function of the proteins to which it is fused. Hence, the characteristics of eqFP611 indicate that it is an ideal starting point for developing novel far-red FPs that overcome these limitations.

The optimization process of eqFP611 has been propelled through mutational analysis, leading to the identification of multiple variants [37]. Such variants, including RFP630, RFP637, and RFP639, are characterized by redder emission peaks due to the chromophore conversion from trans to cis conformation [62]. Dimeric variants share similar spectra and high brightness with eqFP611, but their folding efficiency at 37 °C remains compromised [63]. Conversely, the monomeric version of eqFP611, designated mRuby or meqFP611, demonstrates slightly blue-shifted spectra and enhanced brightness relative to eqFP611 (Table 1) [38]. When employed as a fusion tag, mRuby provides accurate subcellular localization information of fusion proteins and enables the examination of co-localization relationships within living cells [64]. Collectively, these derivatives of eqFP611 introduce a diverse range of options to address the intricate requirements of biological research under varied experimental conditions.

Similar to eqFP611, eqFP578 is a GFP-like protein isolated from the sea anemone *Entacmaea quadricolor* (Table 1) [39]. Sequence alignment reveals that eqFP578 and eqFP611 share 76% homology, indicating that eqFP578 contains the same unique chromophore, which contributes to its highly fluorescent characteristics. Coupled with its ability to mature effectively at physiological temperatures and its reduced tendency of oligomerization, eqFP578 represents a valuable template for the development of enhanced, red-shifted, and monomeric far-red fluorescent proteins.

**Figure 4:**
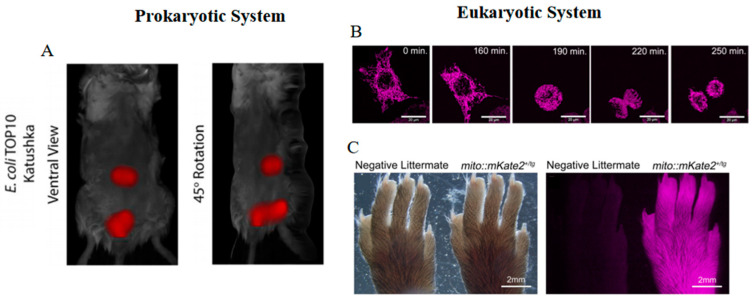
(**A**) Images of Katushka-labeled *E. coli* TOP10 bacteria in mice [16] (reproduced with permission from Copyright 2019, Springer Nature). (**B**) Time-lapse images of mito::mKate2 in Hela cells [67]. (**C**) Images of mito::mKate2 in hind paw of CAG-mito::mKate2^+^ transgenic founder progeny as compared to a negative littermate [67]. The images in (**B**,**C**) were reproduced with permission from Copyright 2018, Wiley.

Katushka2S is a bright dimeric variant of eqFP578 (Table 1) [40]. The bright fluorescence facilitates Katushka2S’s robust and stable expression within bacterial systems. This helps Katushka2S to serve as a DNA damage reporter for monitoring translation blockage upon the induction of antibacterial agents [65,66]. Katushka is another dimeric variant of eqFP578 (Table 1) [41]. Given its bright fluorescence, fast maturation, and stable photostability, Katushka emerges as a satisfactory fluorescent marker for in vivo biomolecule tracking. Utilization of Katushka-labeled *E. coli* expressed in the murine intestine allows for spatial and temporal documentation, alongside quantification in a three-dimensional framework (Figure 4A) [16].

**Figure 5:**
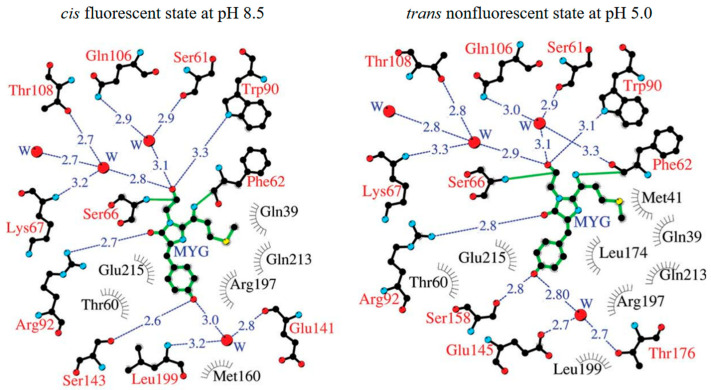
Structure diagrams of Katushka in a cis fluorescent state at pH 8.5 and in a trans nonfluorescent state at pH 5.0. Hydrogen bonds are shown as blue dashed lines, water (W) is shown as red spheres, and van der Waals contacts are shown as black “eyelashes” [68] (reproduced with permission from Copyright 2011, Wiley).

However, a crystallographic study suggests that the spectral property of Katushka is pH dependent and achieved by pH-induced cis-trans isomerization of the chromophore (Figure 5) [68]. When the pH decreases to below 8, the far-red fluorescence of Katushka starts to decline and reaches zero at pH 4.5. tdKatushka2 is a tandem derivative of Katushka (Table 1). Its extra brightness and high photostability render tdKatushka2 amenable for integration with diverse cellular structures [42]. However, the large size of tdKatushka2 might levy deleterious effects on the structural integrity and functional efficacy of the fusion proteins.

mKate, a true monomer variant of Katushka, remains a monomeric structure even at extremely high concentrations, up to 10 mg/mL, and exhibits spectral characteristics identical to those of Katushka (Table 1) [41]. Crystal structure analysis illustrates that mKate undergoes pH-induced cis-trans isomerization similar to Katushka, which leads to diminished fluorescence at lower pH levels [69]. Subsequent in vitro analyses demonstrated the relatively dim brightness and long maturation time, which impedes its utility in bioimaging applications. Therefore, multiple rounds of mutagenesis were conducted to enhance its properties, yielding several improved variants such as mKate2, mKate S158A (also known as mLumin), and mKate S158C.

Due to the high EC, mKate S158A and mKate S158C exhibit significantly greater brightness than mKate, achieving nearly 50% of the brightness of eqFP578 (Table 1) [43]. Unlike mKate S158A, the photobleaching of mKate S158C follows a monoexponential decay, indicative of relatively straightforward kinetics, thus facilitating its use in quantitative time-lapse imaging studies [30]. On the other hand, mKate2 displays similar spectral properties, enhanced photostability, and increased brightness when compared to mKate (Table 1) [42]. This is attributed to a higher proportion of cis conformers that stabilize the chromophore in its luminous state. mKate2 also emits pH-resistant far-red fluorescence in vivo and has been engineered as a pH indicator for monitoring the maturation stages of autophagosomes [70]. Moreover, mKate2 exhibits exceptional biocompatibility, evidenced by its successful application in the genetic or protein labeling of eukaryotic cells and bacterial systems. The development of mito::mKate2 enables intricate observation of mitochondrial architecture, distribution, and dynamism in living cells and transgenic models (Figure 4B,C) [67]. Additionally, the conjugation of mKate2 to the voltage-gated potassium channel Kv1.3 (mKate2-Kv1.3) yields stable and bright fluorescence in mammalian cells, supporting studies of subcellular distribution [71]. Under nanaerobic conditions, mKate2 outperforms DsRed2 and TagRFP in generating the highest far-red fluorescence in *B. fragilis* [72]. However, mKate2 displays a balance between monomeric and dimeric states under high-performance liquid chromatography (HPLC) analysis, which may affect its performance in specific fusion protein constructs [30].

FusionRed has been identified as the purely monomeric version of mKate2 (Table 1) [45]. Structure analyses have elucidated that FusionRed is subject to two independent pathways of post-translational modifications: 60% of its tripeptide undergoes maturation into the conventional red MYG chromophore, while the remaining 40% is characterized by peptide bond hydrolysis. Impressively, both pathways converge in activating the same catalytic residues through a series of subsequent transformations [73]. This intricate post-translational modification phenomenon significantly contributes to the extended maturation half-life of FusionRed, which is prolonged to 130 min. Despite these complexities, FusionRed retains its desirable expression levels and minimal toxicity, making it an optimal candidate for elucidating the spatial distribution of fusion proteins within live cells and rat brains [74,75]. To enhance the optical properties of FusionRed, variants such as FR-M/Q/V and FR-MQV have been engineered [76,77]. FR-M and FR-MQV demonstrate a two-fold and five-fold enhancement in brightness within HeLa cells, respectively, when compared to FusionRed. Additionally, FR-MQ is distinguished by its superior application potential in single-molecule localization microscopy (SMLM), attributed to its enhanced rate of dark state conversion coupled with a longer ground state recovery lifetime [78]. The high brightness and minimal toxicity establish FusionRed and its derivatives as indispensable tools for live-cell imaging.

On the other hand, a novel class of variants has emerged from the foundational mKate template, including Neptune, mNeptune1, mNeptune 2, and mNeptune 2.5. This progression suggests a tailored optimization geared toward enhancing the fluorescent properties and application-specific utility of these proteins in various experimental contexts.

**Figure 6:**
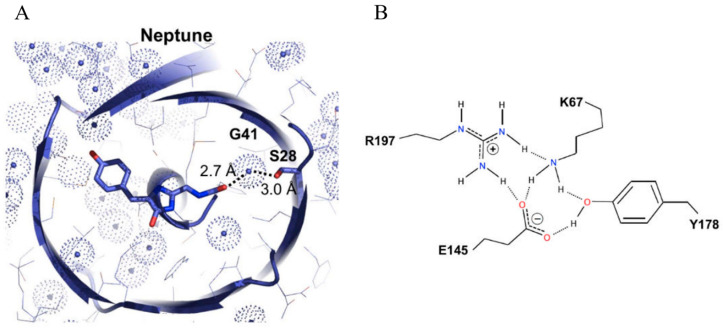
(**A**) Structure diagrams of the Neptune chromophore. The conjugated π system of the chromophore and side chain changes are shown in stick representation with nitrogen in blue and oxygen in red, and the van der Waals surfaces of water oxygen atoms are depicted as dotted spheres colored light blue [30]. (**B**) Detailed model of the hydrogen-bonding network. Hydrogen atoms are attached to donors with solid lines and to acceptors with dotted lines [30]. The images in (**A**,**B**) were reproduced with permission from Copyright 2009, Elsevier.

Neptune is characterized by its redder excitation/emission peaks at 600/650 nm (Table 1) [30]. Analysis of its crystal structure reveals that both the enhanced chromophore coplanarity of its chromophore and the additional hydrogen bond with the acylimine oxygen contribute to the longer wavelengths (Figure 6). With the red-shifted spectra and the fast maturation speed, Neptune demonstrates notable utility for applications under 633 nm laser excitation, particularly for deep-tissue imaging in live organisms. However, an equilibrium between monomeric and dimeric forms at low concentrations has been observed in Neptune, which could potentially affect its efficacy in specific contexts. To address this limitation, a monomeric variant, known as mNeptune, has been developed. mNeptune inherits identical spectral characteristics, high brightness, and rapid maturation speed from Neptune (Table 1) [30]. In applications such as the bimolecular fluorescence complementation (BiFC) system, split mNeptune demonstrates exceptional performance by yielding a marked contrast between the positive and negative groups [79]. Moreover, cell lines expressing mNeptune manifest stable and intense fluorescence, thereby increasing their utility in probing the dynamics of cancer cells under diverse stimuli [22].

The derivatives mNeptune2 and mNeptune2.5 inherit similar spectra from mNeptune (Table 1) [44]. Furthermore, mNeptune2 exhibits roughly 20% brighter fluorescence than mNeptune both in vitro and in vivo, while mNeptune2.5 is 54% brighter than mNeptune in vitro. Among monomeric far-red emitting FPs, mNeptune2 stands out as the superior option for in vivo stimulated Emission Depletion (STED) microscopy owing to its high QY and photostability. This enables elaborate exploration of protein function in live organisms at a super-resolution [80]. From mNeptune2, Crimson was obtained through structure-directed chromophore mutagenesis, displaying nearly 51% increased brightness compared to mNeptune2, despite a 33 nm blue-shift in its emission peak (Table 1) [46]. Importantly, Crimson demonstrates diminished toxicity, a reduced tendency toward aggregation, and an exceptional capacity for long-term tracking. These characteristics render it especially useful for labeling intricate structures such as plasma membranes in neurons [81]. The combination of these advantageous features emphasizes the importance of Neptune and its derivatives in the advancement of biotechnological and medical research.

To sum up, the unique chromophore of eqFP611 and eqFP578 promises them redder spectra and higher brightness, making them brilliant starting points for developing enhanced far-red FPs. eqFP611 variants display longer emission wavelengths, extended maturation temperatures, increased bright fluorescence, and decreased aggregation tendency. These modifications expand the utility of eqFP611 variants, rendering them more suitable for in vivo imaging. The eqFP578 variants offer superior properties over DsRed variants, including enhanced fluorescence intensity, reduced maturation half-time, better photostability, and increased resistance to pH changes. These advantageous properties allow eqFP578 variants to be utilized in a diverse range of applications, such as genetically encoded biosensors, the establishment of stable expression cell lines or animal models, high-throughput screening systems for small molecules, and three-dimensional multi-color fluorescent imaging.

### 2.3. Far Red Fluorescent Proteins from Other Species

HcRed originates from a GFP-like non-fluorescent chromoprotein (CP) isolated from the sea anemone *Heteractis crispa* (Table 1) [48]. To confer fluorescent properties on HcRed, a single amino acid substitution (C148S) was employed to introduce an acylimine into the chromophore. Crystal structure studies demonstrate that HcRed appears in dimer form over a range of concentrations in solution, accommodating both trans and cis conformers within the same protein molecule [82]. Interestingly, the isomerization mechanism from trans-to-cis leads HcRed fluorescent only in its dimer form [83,84,85]. Moreover, HcRed shows vulnerability to direct quenching by copper ions (Cu^2+^) through the formation of a copper–protein complex [86]. Coupled with its diminished brightness and optical instability, HcRed is restricted to an ideal fusion tag. Although the monomeric variants of HcRed, mGinger1, and mGinger2, have been engineered, these variants continue to be hampered by terrible fluorescence brightness and slow maturation rates, limiting their utility in cellular biological imaging [47].

Similar to HcRed, Keima was also engineered from a GFP-like non-fluorescent CP cloned from the stony coral *Montipora* sp., and semi-random mutagenesis was employed to efficiently evolve CP into a functional fluorescent protein [49]. Analytical equilibrium ultracentrifugation analysis has indicated that Keima predominantly forms a homotetrameric complex. Subsequently, dimeric (dKeima) and monomeric (mKeima) variants have been engineered. These variants are distinguished by a notable large Stokes shift and exhibit similar spectral profiles, with excitation and emission peaks at 440 nm and 616 nm, respectively, except for mKeima where the emission peak is slightly red-shifted by 4 nm (Table 1). Interestingly, Keima possesses a bimodal excitation spectrum with an additional peak at 586 nm, which help Keima in exhibiting variable fluorescence colors in a pH-dependent manner, making it a valuable tool for pH indication [87,88]. At a neutral pH, the predominant trans conformation of the chromophore is associated with green fluorescence. Conversely, at low pH, the ascendant cis conformation results in red fluorescence due to the ionized state of the chromophore’s phenolic hydroxyl moiety. Despite their low EC and QY, Keima and its variants have demonstrated robust fluorescence in living cells, thus ensuring their efficacy as fusion tags. Notably, mKeima is particularly advantageous as a precursor for pH indicators due to its pH-sensitive fluorescence color change, offering a potent tool for the exploration of pH dynamics within biological systems.

Based on the observation of co-localization between pink-pigmented lesions in diseased or damaged tissues and the bright red fluorescence in *Porites lobate*, plobRFP was found to be a natural red fluorescent protein with a far-red emission peak at 614 nm (Table 1) [28]. Sequence alignment reveals that plobRFP shares limited similarity with other natural fluorescent proteins, possessing only a 20% sequence identity with av-GFP. However, plobRFP retains all typical secondary structures found in GFP-like proteins, including 4 α-helices, 11 β-sheets, and a tripeptide chromophore that incorporates conserved amino acids YG [89,90]. plobRFP demonstrates a remarkably high molar EC of 84,000 M^−1^ cm^−1^ and a high QY of 0.74, making it the brightest natural far-red FP identified to date (Table 1). Furthermore, plobRFP exhibits exceptional pH resistance, achieving its highest fluorescence intensity at pH 3.5 and maintaining over 70% of this maximum at pH 2.5. Despite its superior optical properties, plobRFP tends to form a tetramer and shows a pronounced tendency for aggregation, which could negatively affect both the folding process and functional integrity of the fused protein.

Above all, the development of FPs like HcRed, Keima, and plobRFP illustrates the diverse potentials and challenges associated with using these molecules as tools in biological research. HcRed and its variants exhibit a significant emission redshift, which permits deeper tissue imaging. Keima and its variants could change fluorescence color in response to different pH levels, making it particularly useful as a dynamic pH indicator within living cells. plobRFP stands out due to its strong pH resistance and remarkable brightness. Future developments may focus on further improving stability, brightness, and maturation rates while reducing tendencies toward aggregation to fully unlock the potential of these and other fluorescent proteins in life sciences.

## 3. Far-Red FP-Based Biosensors

The beneficial properties of far-red FPs, such as red-shifted spectra, bright fluorescence, and deep penetration, collectively demonstrate their reliability in various bioengineering techniques. The tolerance of far-red FPs to insertions and fusions suggests their versatility in the development of biosensors that respond to specific biological stimuli and protein–protein interactions. Initially, these proteins can be fused with sensory parts to facilitate Förster resonance energy transfer (FRET) signaling. Alternatively, the reconstitution of far-red FP assemblies offers a novel approach for split- and circular permutation-based biosensors and reporters. Furthermore, the exceptional pH stability of far-red FPs renders them excellent candidates for usage as pH indicators, affirming their utility in a broad range of biological contexts.

### 3.1. FRET-Based Biosensors

FRET-based biosensors have been extensively employed to visualize crucial molecules involved in cellular signaling and to monitor PPIs in vivo [91]. These biosensors typically consist of a donor and acceptor, connected by a linker that contains two distinct molecule-binding domains. When the target molecule interacts with the biosensor, the FRET FP pairs are brought into close proximity, resulting in efficient FRET signals. Compared to traditional fluorophore pairs within the visible spectrum, far-red FP-based FRET fluorophore pairs provide substantial benefits for multi-color imaging and deep-tissue imaging due to their red-shifted spectra. These systems are capable of delivering clear images with higher resolution and greater accuracy, free from the interference of autofluorescence or light scattering, thus offering a distinct advantage in detailed cellular studies.

**Figure 7:**
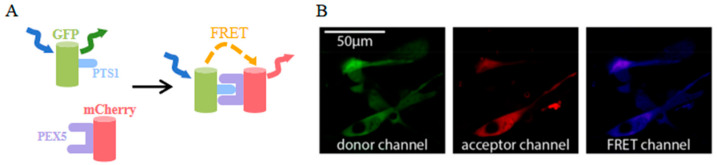
(**A**) Scheme of FRET between EGFP-PTS1 and mCherry-PEX5 [93]. (**B**) Images of EGFP-PTS1 and mCherry-PEX5 in pex5^−/−^ cells [93]. The images in (**A**,**B**) were reproduced with permission from Copyright 2020, Multidisciplinary Digital Publishing Institute.

mCherry was utilized as a FRET acceptor in a novel application of 3-filter FRET measurement techniques [92]. In addition to providing visual insights into interaction dynamics similar to traditional FRET pairs, this innovative mCherry-based FRET approach also allows for the extraction of quantitative measurements such as the interaction stoichiometry or the apparent affinity of the binding partners. The validation of this new measurement approach was assessed by studying the interaction between different type-1 peroxisomal targeting signals (PTS1) and their soluble receptor peroxin 5 (PEX5) (Figure 7A) [93]. In cells lacking endogenous PEX5, EGFP-PTS1, and mCherry-PEX5 co-localize across the cytosol, and the FRET signal is effectively detected (Figure 7B). Significantly, this technique allows for the evaluation of how sequence elements adjacent to the core tripeptide within PTS1 motifs influence the interaction strength between PTS1 and PEX5.

**Figure 8:**
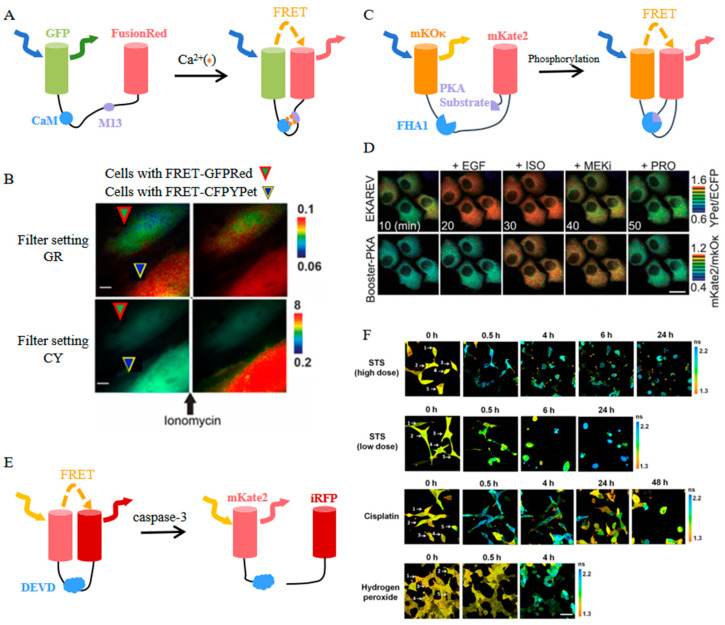
(**A**) Scheme of FRET-GFPRed [94]. (**B**) Images of FRET-GFPRed and FRET-CFPYPet in two Hela cells of one field before and after ionomycin stimulation [94]. The images in (**A**,**B**) were reproduced with permission from Copyright 2020, Multidisciplinary Digital Publishing Institute. (**C**) Scheme of Booster-PKA [95]. (**D**) Time-lapse images of Booster-PKA and EKAREV in Hela cells before and after treatments with indicated stimulants and inhibitors [95]. The images in (**C**,**D**) were reproduced with permission from Copyright 2020, American Chemical Society. (**E**) Scheme of mKate2-DEVD-iRFP. (**F**) Time-lapse images of the donor mKate2 before (0 h) and after indicated treatments [98] (reproduced with permission from Copyright 2022, Springer Nature), Individual cells in (**F**) are numbered.

EGFP and FusionRed were selected as a FRET fluorophore pair for developing a novel Ca^2+^ sensor referred to as FRET-GFPRed (Figure 8A) [94]. This biosensor consistently exhibits a 15% increase in the FRET/donor ratio in response to various stimuli. Moreover, the spectral compatibility of FRET-GFPRed and FRET-CFPYPet (a typical FRET-based Ca^2+^ biosensor with ECFP and YPet) enables dual-FRET imaging of Ca^2+^ signals from two separate cells (Figure 8B).

Through a rigorous screening process for the optimal Förster distance, mKOκ and mKate2 were carefully chosen to form a FRET biosensor backbone named Booster (Figure 8C) [95]. Booster-based biosensors designed for detecting JNK, ROCK, ERK, and PKA exhibit significantly elevated response signals compared to their CFP/YFP-based counterparts [95]. Furthermore, the versatility of Booster extends to its application in dual FRET imaging techniques, enabling the concurrent monitoring of the activities of two distinct protein kinases when paired with a conventional CFP- and YFP-based FRET biosensor (Figure 8D) [95].

With the red-shifted spectra, the mRuby-based FRET pair can be adapted for the visualization of tripartite protein interactions, which is unachievable for the traditional CFP/YFP FRET pair [96]. This system has been achieved by a bioluminescence resonance energy transfer (BRET) pair composed of nanoLuc luciferase (NL) and superfolder green fluorescent protein (sfGFP), along with a FRET pair comprising mRuby and sfGFP. The sequential energy transfer from NL to sfGFP, and subsequently to mRuby, facilitates the observation of the co-assembly of three distinct peptides, providing a new approach for the investigation of intricate biological processes.

Besides functioning as an acceptor, far-red FPs like mKate2 can also act as donors in FRET pairs. A genetically encoded sensor for caspase-3 activity was engineered using mKate2 linked to the near-infrared fluorescent protein iRFP through a linker containing a caspase-3 cleavage site DEVD (Figure 8E) [97]. Upon activation of caspase-3, mKate2-DEVD-iRFP exhibits an increase in mKate2 fluorescence intensity and the FRET donor/acceptor ratio. With this characteristic, the dynamic of caspase-3 activity in cancer cells could be monitored during the apoptotic process at the level of individual cells, which facilitates enhancing the efficacy of antitumor drugs and overcoming apoptotic resistance (Figure 8F) [98].

Far-red FP-based FRET biosensors have revolutionized the visualization of cellular signaling molecules and PPIs in live organisms, especially in multi-color imaging. Notably, mCherry has emerged as a FRET acceptor in a novel three-filter FRET technique, which facilitates the study of interaction dynamics and enables quantitative analysis of binding partner affinities. A FusionRed-based calcium sensor has demonstrated an enhanced FRET ratio and dual-color imaging of calcium signals. The Booster backbone, incorporating mKOκ and mKate2, has yielded improved responses for visualizing kinase activity. Moreover, the mKate2-based caspase-3 biosensor highlights the utility of far-red FPs in multi-color imaging and the exploration of intricate biological processes. Additionally, far-red FP-based FRET biosensors, along with the BRET pair, have been extended to facilitate the study of interactions among three proteins, thereby broadening the tools available for investigating complex biological mechanisms.

### 3.2. Split Protein-Based Biosensors

Split fluorescent proteins represent an invaluable tool for monitoring PPIs via the BiFC system [99]. The intrinsic characteristics of far-red FPs, notably their capacity to penetrate skin and display bright fluorescence, endow far-red FP-BiFC systems with distinct advantages for exploring the spatiotemporal dynamics of PPIs in vivo within deep tissues.

mKate was initially utilized in the BiFC system through strategic splitting within its loop regions [43]. When assayed with the basic leucine zipper domains of Fos (bFos) and Jun (bJun), the resultant domains exhibited split properties. However, the practical application of the mKate-BiFC system has been limited by its inherent instability and relatively low brightness, leading to an elevated false-positive rate and a reduced signal-to-noise ratio.

**Figure 9:**
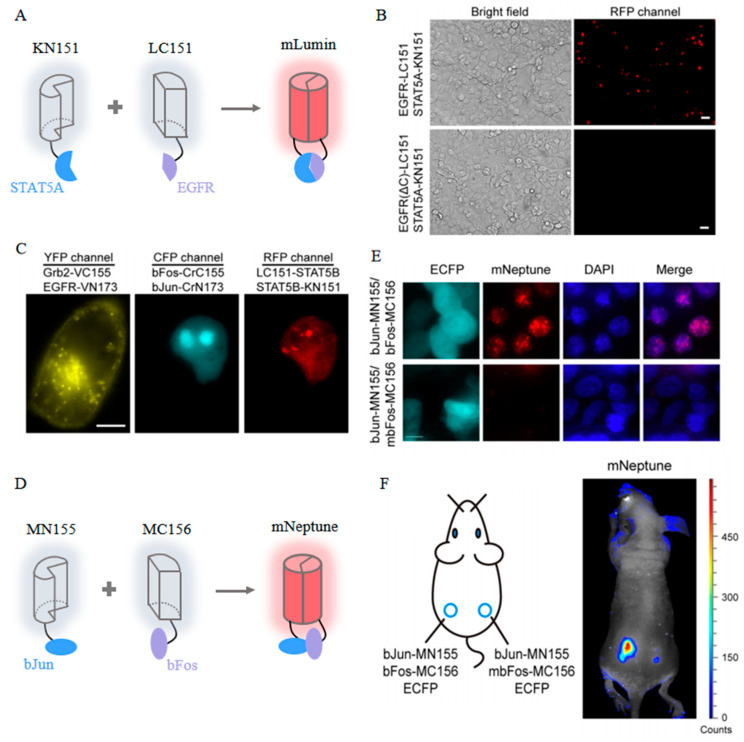
(**A**) Scheme of the mLumin-BiFC system [43]. (**B**) Images of mLumin-BiFC signals in COS-7 cells with indicated proteins. Scale bar: 20 μm [43]. (**C**) Images of three pairs of protein interactions in the same living cells with indicated BiFC systems. Scale bar: 10 μm [43]. The images in (**A**–**C**) were reproduced with permission from Copyright 2009, Elsevier. (**D**) Scheme of the mNeptune-BiFC system [101]. (**E**) Images of mNeptune-BiFC signals in live cells with indicated proteins [101]. (**F**) Images of mNeptune-BiFC signals in live mice injected subcutaneously with indicated cells [101]. The images in (**D**–**F**) were reproduced with permission from Copyright 2014, Oxford University Press.

To address the above issues, site-saturation mutagenesis was undertaken, resulting in mLumin and mKate-S158C [43]. Both the mLumin- and mKate-S158C-BiFC systems demonstrated significantly improved red fluorescence, exhibiting 1.5- and 1.4-fold increases in BiFC efficiency, respectively. Moreover, the mLumin-BiFC system proves for the first time that epidermal growth factor receptor (EGFR) directly interacts with STAT5 (signal transducers and activators of transcription) in living cells, which enriches the EGFR signaling pathway and provides new possibilities for the treatment of related diseases (Figure 9A,B). Furthermore, the spectral distinctiveness of mLumin from Cerulean and Venus enables the simultaneous visualization of three distinct pairs of PPIs within the same cellular context (Figure 9C). Additionally, the efficacy of the mLumin-BiFC system can be further enhanced by incorporating a bicistronic expression vector, which minimizes the spontaneous association of FP fragments [100]. This advanced modification in the mLumin-BiFC system results in a remarkable 25-fold increase in contrast regarding BiFC efficiency between positive and negative controls.

mNeptune splits at the 155th amino acid residue located within the loops of its barrel-like structure [101]. In comparison to BiFC systems operating within the visible spectrum, mNeptune-BiFC systems significantly reduce the interference of light absorption and enhance excitation efficiency. In cells co-expressing bJun-MN155 and bFos-MC156, the intensity of the red BiFC signals is approximately 3.5-fold greater than that observed in negative control cells (Figure 9D,E). Furthermore, this enhancement in fluorescence signal is consistently replicated in vivo through the injection of murine models with both positive and negative cell lines at different anatomical sites (Figure 9F).

Split far-red FPs play a pivotal role in facilitating PPIs, offering the advantage of noninvasive imaging in live organisms. Although the mKate-BiFC system has been limited by issues of instability and low fluorescence intensity, the new BiFC systems based on mLumin and mKate-S158C prove their potential in in vivo imaging by exhibiting enhanced brightness and stability. Additionally, the red-shifted spectra allow the mLumin-BiFC system to simultaneously visualize multiple PPIs. Furthermore, the mNeptune-BiFC system displays significant BiFC efficiency in live mice, illustrating the utility of this system for the deep-tissue analysis of PPIs.

### 3.3. Circularly Permuted Protein-Based Biosensors

Circularly permuted fluorescent proteins (cpFPs) are a versatile group of biosensors that enable the observation of various intracellular processes [102]. In cpFPs, the original N- and C-termini are fused by a peptide linker, positioning the new N- and C-termini in close proximity to the chromophore. This structural modification imparts greater mobility to the fluorescent protein compared to its native variant, thus enhancing the lability of its spectral characteristics. The common approach for creating genetically encoded biosensors involves integrating cpFP into a flexible region of a sensory domain or between two interacting domains [103,104] or splitting cpFP into two fragments using the sensory domain as the interface, with the new termini left free to interact and reassemble into a mature chromophore [105]. Conformational rearrangements of the sensory domain induced by ligand interactions or changes in cellular parameters are transferred to the cpFP, thereby altering the chromophore environment and enabling the detection of target molecules. Among various cpFP-based biosensors, circularly permuted far-red FP-based biosensors are particularly effective in visualizing molecules in vivo, especially in deep tissue, due to their redder excitation and emission spectra.

**Figure 10:**
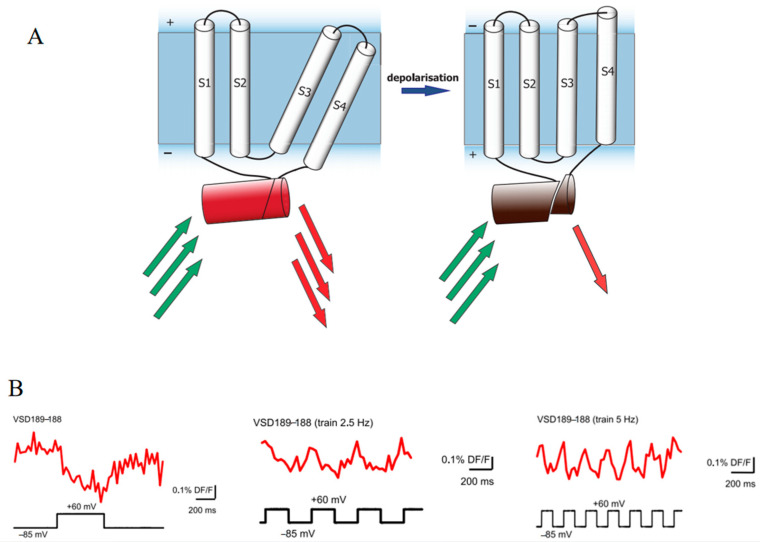
(**A**) Scheme of the cpFusionRed-based voltage sensor. S1–S4 are transmembrane voltage-sensitive domains (VSDs). The red barrel is FusionRed. The green and red arrows represent excitation and emission light, respectively [105]. (**B**) Images of fluorescence changes to single voltage steps and trains of 2.5 Hz and 5 Hz in voltage-clamped PC12 cells [105]. The images in (**A**,**B**) were reproduced with permission from Copyright 2017, Public Library of Science.

Circularly permuted FusionRed (cpFusionRed) has been integrated into fluorescent protein-based voltage sensors with insertion-into-circular permutant topology [105]. In this biosensor, the FusionRed polypeptide chain is divided into two fragments by the voltage-sensitive domain (VSD). Changes in transmembrane potential induce substantial conformational shifts in the VSD, thereby altering the relative spatial positions of its termini and resulting in fluorescence changes in the biosensor (Figure 10A). This innovative design allows for the direct observation of electrical activity within cells and tissues. Notably, the fluorescent signal of this biosensor demonstrates a circularly consistent alteration in response to a sequence of stimulations composed of repetitive voltage steps (Figure 10B). Importantly, the time constant tau (obtained from exponential fits) and the amplitude of ON-responses of this biosensor are comparable with those of a previously reported successful voltage-sensitive biosensor. Furthermore, the emission of far-red light significantly enhances its penetration capabilities, thereby enabling the reliable recording of signals from the body surface of an intact organism.

Circularly permuted mRuby has been utilized in the development of genetically encoded calcium indicators (GECIs) [106]. These novel red GECIs demonstrate calcium sensitivity comparable to that of traditional GFP-based GECIs and exhibit superior performance in detecting and quantifying neural activity across a broad spectrum of organisms, including cultured neurons, mice, *Drosophila*, zebrafish, and *C. elegans*. Additionally, red GECIs enhance deep-tissue imaging capabilities, enable dual-color imaging in conjunction with GFP-based reporters, and facilitate the integration of optogenetics with calcium imaging.

Circularly permuted far-red FPs are significant in monitoring molecules in deep tissue. The first fluorescent voltage sensor using cpFusionRed exhibits improved tissue penetration capabilities and comparable sensitivity compared to previous biosensors. Red GECIs offer better performance in living organisms, deep-tissue and dual-color imaging, and optogenetic integration.

### 3.4. pH Indicator

The inherent pH stability of far-red FPs enhances their utility for the real-time monitoring of pH fluctuations within biological systems, presenting a novel approach for in vivo pH testing. Unlike other techniques previously discussed, this approach is achieved by the outstanding pH stability in acidic organelles or the pH-sensitive dual-excitation ratiometric properties, without altering the structural integrity of the far-red FPs.

**Figure 11:**
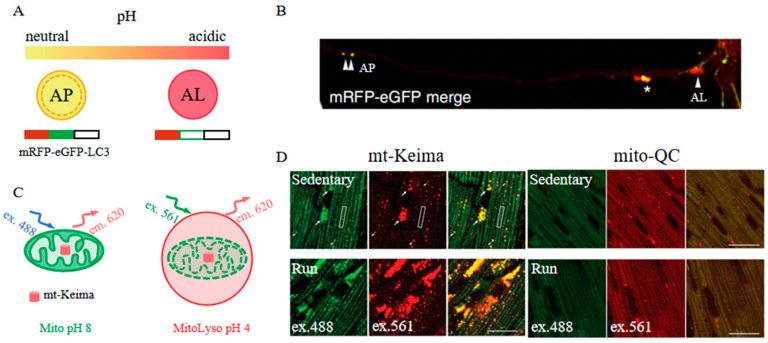
(**A**) Scheme of mRFP-eGFP-LC3 [51]. (**B**) Images of mRFP-eGFP-LC3 in primary neurons. APs (double arrowheads), ALs (arrowhead), and pa-ALs (asterisk) are shown as indicated [51]. The images in (**A**,**B**) were reproduced with permission from Copyright 2022, Springer Nature. (**C**) Scheme of mt-Keima [115]. (**D**) Images of mt-Keima and mito-QC in the mouse heart following the exhaustive exercise protocol. Mitolysosomes (white arrows) and mitochondria (white boxes) are shown as indicated. Scale bar: 20 μm [115]. The images in (**C**,**D**) were reproduced with permission from Copyright 2021, Taylor & Francis.

The mRFP-eGFP-LC3 has been developed as a novel pH indicator through the integration of mRFP, eGFP (a pH-sensitive fluorescent protein), and LC3 (an autophagy adaptor protein), facilitating the study of the autophagy maturation process (Figure 11A) [51]. Under physiological conditions, mRFP-GFP-LC3 emits a yellow-green fluorescence because both mRFP and eGFP fluoresce at this pH. However, as autophagosomes (APs) fuse with lysosomes to form autolysosomes (ALs), the increased acidity of the environment leads to the quenching of eGFP fluorescence, resulting in a shift to orange and subsequently red fluorescence, indicative of the progressive maturation stages of ALs (Figure 11B). This characteristic renders mRFP-eGFP-LC3 a valuable tool for investigating mechanisms of drug resistance and the pathogenesis of diseases related to autophagy [107,108,109]. Similarly, mCherry, another fluorescent protein that maintains stable fluorescence in acidic conditions, is employed with eGFP-LC3 to explore autophagic flux in living cells [110].

mKate2 has been incorporated into building an AL maturation indicator, named pHlurorin-mKate2-human LC3, in which pHluorin is a pH-sensitive green FP. pHlurorin-mKate2-human LC3 has been successfully employed to track autophagic flux in cells under starvation [111,112]. Likewise, a genetically encoded pH indicator based on FusionRed has been shown to monitor intracellular pH fluctuations in *Drosophila* [113].

An alternative mechanism by which far-red FPs serve as pH indicators is exemplified by Keima [114]. Keima is distinguished by its pH-sensitive dual-excitation ratiometric properties and its notable resistance to degradation within lysosomes. At the physiological pH in mitochondria (pH 8.0), the shorter-wavelength excitation of Keima is more dominant, allowing for the capture of emission signals by a 458 nm laser excitation. Conversely, when mitochondria become damaged and are sequestered within the acidic lysosome (pH 4.5), Keima undergoes a gradual shift to longer-wavelength excitation, obtaining effective emission signals through a 561-nm laser excitation (Figure 11C). Based on this, mt-Keima is utilized for reporting mitophagy and shows enhanced sensitivity compared to the conventional mitophagy reporter mito-QC [115]. Using an exhaustive exercise (EE) protocol that activates PINK1-PPKN mitophagy, mt-Keima exhibits an increase in mitolysosomal area with EE, indicating the activation of mitophagy; however, mito-QC expression is stable under both 458 and 561 nm laser excitation between the run group and sedentary group, suggesting that no phenomenon of mitophagy is observed (Figure 11D). This sensitivity has proven invaluable in exploring the potential pathogenesis of diseases linked to dysfunctional mitophagy, including obesity-associated cardiomyopathy, hepatocellular carcinoma, and pancreatic β-cell dysfunction [116,117,118]. Furthermore, Keima demonstrates its value in the investigation of other autophagic processes, including ribophagy and lysophagy [119,120,121]. This versatility highlights Keima’s potential as a powerful tool in the broader study of autophagy and its implications in health and disease.

The inherent ability for stable expression under acidic conditions positions far-red FPs as optimal pH indicators for autophagy studies. The probe mRFP-eGFP-LC3 elucidates autophagic maturation by displaying a color transition from yellow–green to red. Similarly, variants such as mCherry, mKate2, and FusionRed are strategically utilized to monitor autophagic flux in vivo. In addition, Keima distinguishes itself as a pH indicator by its pH-sensitive dual-excitation wavelengths. Indicators based on Keima demonstrate superior efficiency in monitoring the mitophagy process when compared to conventional mitophagy reporters. Moreover, the versatility of Keima extends beyond its application in mitophagy, encompassing the study of ribophagy and lysophagy as well.

## 4. Far-Red FPs in Multi-Color Imaging

Multi-color imaging facilitates the simultaneous visualization of multiple components, enhancing the understanding of complex biological interactions and structures. In multi-color imaging, different FPs can be excited using different excitation wavelengths, and their emitted fluorescence signals are detected at corresponding emission wavelengths. When the gene encoding a fluorescent protein is fused with the gene coding the target protein, the resulting fusion protein inherits the fluorescent properties of the FP, and its subcellular localization can be observed via fluorescence microscopy. With the red-shifted emission spectra, far-red FPs have expanded the fluorescent protein spectrum beyond the traditional GFP range. This expansion provides far-red FPs for three-, four-, or even six-color imaging with other spectrally discrete FPs.

**Figure 12:**
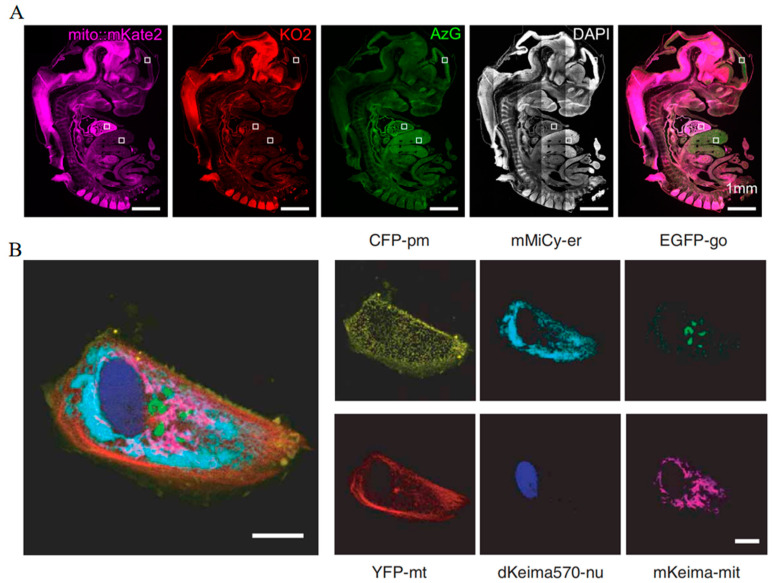
(**A**) Images of fluorescent signals in the mKate2, KO2, and AzG channels throughout the mice embryo. The higher magnification images within the white boxes are shown in the original article. Scale bar: 1 mm [67] (reproduced with permission from Copyright 2018, Wiley). (**B**) Images of fluorescent signals in the CFP, mMiCy, EGFP, YFP, dKEIMA570, and mKeima channels in one Vero cell. Scale bar: 10 μm [49] (reproduced with permission from Copyright 2006, Springer Nature).

With distinct spectral separation, mito::mKate2 and cell cycle reporters including Fucci-KO2 and Fucci-AzG exhibit clear and independent fluorescent signals in mouse embryos, which contributes to tracking mitochondrial dynamics at different cell cycle stages (Figure 12A) [67]. Additionally, mKeima, utilized for six-color imaging, allows for the simultaneous assessment of six distinct subcellular structures within a single Vero cell, enabling a detailed examination of the spatial relationships and interactions among these organelles (Figure 12B) [49]. Moreover, the utilization of mCherry in three-channel imaging has been instrumental in delineating the subcellular localization of targeted proteins, thereby offering information on their functional dynamics within cells [122]. The employment of mRuby specifically labels medial septal GABAergic neurons (MSGNs), facilitating the monitoring of MSGN dynamics in live mice under various stimuli [123].

## 5. Far-Red FPs in Enhanced Resolution Imaging

Fluorescent proteins with red-shifted excitation and emission wavelengths are suitable for sensitive imaging in vivo since they overcome the obstacles of light scattering, tissue absorbance, and autofluorescence. However, the resolution attainable with these proteins is limited. Recent advancements in imaging techniques suggest that far-red FPs could facilitate the achievement of enhanced resolution at deeper tissue depths.

### 5.1. Photoacoustic Imaging

Photoacoustic (PA) imaging combines high spatial and temporal resolution with the deep penetration of ultrasound, enabling detailed visualization of structures within deep biological tissues [124]. In PA imaging, the target within tissues or samples is optically excited by a pulsed laser, causing local transient thermal expansion due to absorbed light energy, thereby generating acoustic pressure waves. These emitted waves propagate through adjacent tissue layers and are subsequently detected by ultrasound (US) transducers.

**Figure 13:**
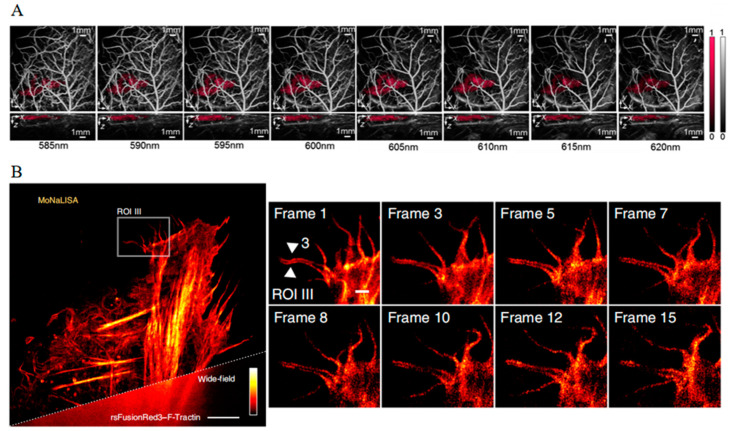
(**A**) Images of NFA-labeled tumor cells in a nude mouse acquired at wavelengths between 585 and 620 nm [125] (reproduced with permission from Copyright 2020, Society of Photo-Optical Instrumentation Engineers). (**B**) Images of rsFusionRed2–F-Tractin in live U2OS cells with MoNaLISA nanoscopy [127] (reproduced with permission from Copyright 2018, Springer Nature).

By employing a modified version of E2-Crimson (NFA), it becomes feasible to distinguish labeled tumor cells from a background of intense vasculature in vivo at a deep depth (Figure 13A) [125]. Additionally, photoacoustic tomography (PAT) studies using E2-Crimson demonstrated a distinct depth advantage in resolving FP in an animal model [126]. Consequently, PA imaging with far-red FPs presents a superior method for deep-tissue analysis, offering unprecedented spatial and temporal resolution alongside enhanced penetration capabilities.

### 5.2. Fluorescence Nanoscopy Imaging

Fluorescence nanoscopy imaging allows for the unprecedented high-resolution visualization of molecular processes and structures at the nanoscale within cells [128]. Fluorescent nanoscopy employs super-resolution imaging techniques such as stimulated emission depletion microscopy (STED) and structured illumination microscopy (SIM) to enhance image resolution and improve imaging quality by minimizing light scattering and background noise. By utilizing reversibly photoswitchable FPs, this technology enables the observation of complex cellular structures at the nanoscale level. These photoswitchable FPs undergo structural changes upon excitation with specific wavelengths of light, altering the intensity or frequency of their fluorescence emission. Through precise control of excitation and emission processes, this modulation can be accurately captured, thereby providing enhanced biological imaging capabilities.

In comparison to reversibly photoswitchable green fluorescent proteins, far-red reversibly photoswitchable fluorescent proteins (rsFPs) can be excited by green–orange light, which not only reduces phototoxic effects but also enables multicolor imaging. The mutant reversibly photoswitchable mCherry, known as rsCherryRev, offers unique optical properties, exhibiting a negative-switch mechanism where yellow light triggers an on-to-off transition, and blue light facilitates the off-to-on switch [129]. Moreover, the employment of rsCherryRev has enabled the visualization of dynamic changes within the complex ER structures at nanoscale resolution in living mammalian cells, which is unachievable with traditional far-field microscopy. However, limitations such as low brightness and unstable photostability deter its broader application. To address these challenges, reversibly photoswitchable FusionRed (rsFusionRed) has been developed [127]. rsFusionRed demonstrates greater brightness, quicker switching kinetics, and more consistent photostability. It is also noteworthy as the first in its class to facilitate switching with milder green light, thereby reducing potential harm to cells. The practical application of rsFusionRed extends to labeling and monitoring the structural dynamics of small actin bundles with an impressive spatial resolution below 80 nm with molecular nanoscale live imaging with sectioning ability (MoNaLISA) nanoscopy (Figure 13B). Additionally, it accurately records the dimensions of vimentin filamentous structures down to a level of 50–70 nm. This degree of precision and capability in live-cell imaging represents a considerable advancement, offering unprecedented insights into the dynamic structural organization at the nanoscale within cellular environments.

## 6. Perspective

Far-red FPs are characterized by their red-shifted excitation and emission spectra, endowing them with the ability of deep tissue penetration, diminished light absorption and scattering, and reduced phototoxicity. With these advantages, far-red FPs allow for the rapid expansion of imaging technologies and provide significant advantages for biological research, notably in the non-invasive observation of cellular and molecular processes in vivo. Far-red FPs also play an essential role in tracking dynamic biological events, labeling cellular components, and studying organismal structures. Their application spans from detailed cellular analysis to whole-body imaging, making them invaluable in disease research, drug discovery, and monitoring therapeutic effects in real time.

Numerous far-red FPs have been engineered through various mutagenesis strategies. The reforming tendency of far-red FPs always moves toward monomeric form, higher brightness, red-shifted spectra, faster maturation, and higher photostability. However, just as no fruit in a grocery store can supplant all others, there is no single best fluorescent protein within the cornucopia. eqFP578-derived far-red FPs generally display brighter fluorescence than their DsRed-derived counterparts at the same excitation wavelengths. For example, mKate is brighter than mCherry, and mNeptune is brighter than mRaspberry. The monomeric series prefer exhibiting red shifting spectra at the cost of reduced brightness, such as mRFP1, which is characterized by a 24 nm redder emission peak but 78% less brightness than DsRed.

The development of new far-red FPs with comprehensive improvement in the aspect of photophysical and biochemical properties is highly desired. Such improved far-red FPs can serve as crucial tools for labeling biomolecules precisely and detecting critical signaling molecules sensitively. This is exceptionally promising for elucidating complex and interconnected cellular processes. The advancements in new far-red FPs considerably augment their potential for attaining high temporal and spatial resolution in multi-color and 3D imaging applications, as well as enabling long-term molecule tracking. Moreover, these proteins may significantly contribute to adjuvant therapies during surgical operations, potentially amplifying the efficacy of treatments, improving disease cure rates, and bolstering patient survival outcomes.

## Data Availability

Data sharing does not apply to this article.
